# Investigating On-Road Lane Maintenance and Speed Regulation in Post-Stroke Driving: A Pilot Case–Control Study

**DOI:** 10.3390/geriatrics6010016

**Published:** 2021-02-09

**Authors:** Heng Zhou, Qian (Chayn) Sun, Alison Blane, Brett Hughes, Torbjörn Falkmer, Jianhong (Cecilia) Xia

**Affiliations:** 1 School of Tourism and Geography Science, Qingdao University, Qingdao 266071, China; heng.zhou@postgrad.curtin.edu.au; 2 School of Earth and Planetary Sciences, Curtin University, Perth 6845, Australia; c.xia@curtin.edu.au; 3 School of Science, RMIT University, Melbourne 3001, Australia; 4 School of Occupational Therapy and Social Work, Curtin University, Perth 6845, Australia; alison.blane@curtin.edu.au (A.B.); T.Falkmer@curtin.edu.au (T.F.); 5 Department of Transport, Perth 6000, Australia; p7safety@gmail.com; 6 School of Health Sciences, Jönköping University, 551 11 Jönköping, Sweden; 7 Rehabilitation Medicine, Department of Medicine and Health Sciences (IMH), Faculty of Health Sciences, Linköping University & Pain and Rehabilitation Centre, UHL, County Council, 581 83 Linköping, Sweden; 8 School of Occupational Therapy, La Trobe University, Melbourne 3083, Australia

**Keywords:** post-stroke drivers, vehicle movement trajectory, standard deviation of lane deviation, speed control

## Abstract

Stroke can adversely affect the coordination and judgement of drivers due to executive dysfunction, which is relatively common in the post-stroke population but often undetected. Quantitatively examining vehicle control performance in post-stroke driving becomes essential to inspect whether and where post-stroke older drivers are risky. To date, it is unclear as to which indicators, such as lane keeping or speed control, can differentiate the driving performance of post-stroke older drivers from that of normal (neurotypical) older drivers. By employing a case–control design using advanced vehicle movement tracking and analysis technology, this pilot study aimed to compare the variations in driving trajectory, lane keeping and speed control between the two groups of older drivers using spatial and statistical techniques. The results showed that the mean standard deviation of lane deviation (SDLD) in post-stroke participants was higher than that of normal participants in complex driving tasks (U-turn and left turn) but almost the same in simple driving tasks (straight line sections). No statistically significant differences were found in the speed control performance. The findings indicate that, although older drivers can still drive as they need to after a stroke, the decline in cognitive abilities still imposes a higher cognitive workload and more effort for post-stroke older drivers. Future studies can investigate post-stroke adults’ driving behaviour at more challenging driving scenarios or design driving intervention programs to improve their executive function in driving.

## 1. Introduction

Driving is an important, autonomous daily activity, which underpins personal mobility in society [1]. It is a vital skill that requires the use of multiple neuropsychological processes such as cognitive, visual, perceptual and data processing abilities [2].

It has been well reported that older adults tend to experience cognitive decline with increased age [3,4,5,6] and this decline can be accentuated by the neural brain damage caused by a stroke [7,8], thereby having a subsequent adverse impact on their driving ability [9]. Although studies show returning to driving post-stroke with rates between 30 and 68% reported in Australia and other countries [10], since the post-stroke population is expected to rise to over 132,000 by 2050 in Australia [11] and driving is still a key aspect of maintaining their independence, the population of post-stroke individuals who wish to return to driving is likely to rapidly increase [12]. In consequence, ensuring a safe return to driving through accurate driving measurements is required, particularly as the older driver population and post-stroke drivers are at an increased risk of a crash and of fatal injuries due to increased frailty [13,14,15].

Currently, driving simulators and on-road driving assessments are the two main methods used to assess driving behaviours, such as lane keeping performance [16,17]. For the simulated driving scenarios, the participants perform a lane maintaining task and their lane deviation is calculated automatically by a driving simulator program [16]. In contrast, on-road assessments involve completing a variety of driving tasks on-road, in a licensed vehicle, and these are considered the “gold standard” because they take place in the real world and are therefore more likely to gain a more accurate representation of drivers’ performance [18,19]. Although preferable as an assessment method, it is hard to detect subtle variations between drivers in an on-road assessment if they do not make any errors as traditional driving assessments rely on subjective observations from driving evaluators. A between-groups study included a post-stroke driver group and a group of similarly aged older control drivers who were observed for their driving behaviours in simulator-based driving scenarios [20], and no differences were found in the amount of the perceived tasks demand required to complete the driving tasks.

Anstey and Wood [2] pointed out that using a fixed driving route for on-road testing is an efficient strategy to examine the participant’s driving performance. Therefore, the optimal approach is to record their driving in naturalistic settings at a microscopic level using a fixed driving route. An advanced global navigation satellite system, such as GPS technology, can be applied to tracking vehicle movements and trajectories, which can be used to ascertain the driver’s driving behaviour on-road assessment.

The driving trajectory and behaviours are both dependent on the position data from the Global Positioning System (GPS), and thus the accuracy and precision of the GPS data become the key points in analysing the driver’s driving trajectory and behaviours. Recently, multiple satellite systems have contributed significantly to global navigation and positioning systems with regard to precision and availability. Generally, the combination of multi-global navigation satellite system (GNSS) receivers can be used to collect and detect a driver’s driving trajectory [21]. It can record the position data of a vehicle to one tenth of a second with a high degree of precision (1 decimetre), which is important for improving driving trajectory detection accuracy. In comparison with single GPS technology, multi-GNSS can provide a better approach to recording more accurate position information since more satellites can be tracked, which will be efficient in tracking the driver’s driving trajectory. To improve the raw position data, corrections can be applied to the recorded position data when the accuracy is particularly important [22]. Specifically, efficient GPS position techniques can be used to correct errors in the position data. For example, real-time kinematic (RTK) can be applied to enhance the accuracy and precision of the position data which can be fitted to track driving trajectories. The RTK technique introduces not only GNSS code pseudo-range measurements to compute its position, and atmospheric errors such as troposphere errors and ionosphere errors could be considered and evaluated [23], it also applies carrier phase measurements, which can provide positions that are orders of magnitude. Therefore, the position data could reach an accuracy level of between a millimetre and a centimetre [22]. By adopting the millimetre to a centimetre measure of the driving trajectory using multi-GNSS RTK technologies, lane keeping performance can be assessed at a high accuracy level in a geographic information system (GIS) platform [22].

The primary aim of this study is to explore the quantitative variations in vehicle control performance between post-stroke and normal older drivers using multi-GNSS and RTK techniques. Based on the solid literature review [17,24], the objectives of this paper are to examine the vehicle control performance in post-stroke driving in comparison with normal drivers’ performance in older drivers through a pilot case–control study and to explore potential indicators of post-stroke driving that can be employed in driving intervention design for this cohort of drivers.

## 2. Materials and Methods

### 2.1. Participants

Participants comprised 14 post-stroke adults (79% male, M = 71.1 years, SD = 6.6) and 14 older drivers who had not had a stroke (57% male, M = 72.9 years, SD = 6.5) to act as a comparison group; all the participants were aged 60 or older. The inclusion criteria for the study participation were that participants held a driving licence valid within Australia, had at least one year of overall driving experience, drove at least twice a week and had access to a fully insured vehicle. Further criteria for the post-stroke cohort were that they had previously been diagnosed with a stroke (either ischemic, haemorrhagic or a transient ischemic attack) and had been cleared to drive by a medical professional. Group selection was based on self-reported data; however, medical records provided by the participant were reviewed to confirm the stroke diagnosis when possible. Participants were excluded if they had been diagnosed with hemianopia, a neurodegenerative disease, such as Parkinson’s disease or dementia, and if they required a wheelchair to get around. Preliminary screening on vision and cognitive conditions was also conducted for all participants to ensure compliance with inclusion criteria using self-report, the Snellen Visual Acuity Chart and the Mini-Mental State Examination (MMSE). Participants were recruited using volunteer sampling techniques such as using local community groups, post-stroke support groups, community newspapers and local radio stations.

### 2.2. Procedure and Data Collection

#### 2.2.1. Procedure

This pilot study employed a case–control design to measure and compare the differences in on-road lane maintenance, trajectory and speed control in post-stroke drivers and age-matched controls using multi-GNSS RTK and GIS technologies.

The on-road driving task was completed within and around Curtin University, Bentley, in Western Australia. The on-road assessment route (Appendix A) was specifically selected to contain intersections, roundabouts, U-turns and traffic lights. Participants were unaware of the route prior to the assessment and were given directions as the route progressed, for example, the drivers were required to notice and react to different speed limits. Additionally, in order to more accurately examine their lane keeping performance, the participants were instructed to maintain the centre line of the lane as much as possible. The whole route was approximately 9 km long, containing various speed limits and traffic scenarios, and took approximately 20 min to complete. Data were collected for the duration of the assessment route and also data from specified points during the route were then processed and mapped using ArcGIS software, following the procedure of Sun et al. [25]. The residual output provided accurate vehicle coordinates used to calculate the standard deviation of lane deviation (SDLD) and speed control across all participants in both groups.

#### 2.2.2. Data Collection

Data collection for this study took place during the normal traffic time. Participants were required to complete an on-road driving task with their own vehicle. A Trimble R10 Multi-GNSS receiver was attached to the roof of the vehicle recording moment-to-moment GPS data at 10 Hz, which records the vehicle positions at every 0.1 s.

To improve the raw position data, corrections were applied to the recorded position data. Following Sun et al. [26], efficient GPS position techniques were used to correct errors in the position data. The real-time kinematic technique (RTK) was applied to enhance the accuracy and precision of the position data, which were fitted to track driving trajectories. By adopting the millimetre to centimetre measure of the driving trajectory using multi-GNSS RTK technologies, lane keeping performance was assessed at a high accuracy level, therefore providing a solid foundation for this comparative study of driving performance in normal and post-stroke older drivers [26].

### 2.3. Driving Performance Measures

#### 2.3.1. Lane Keeping

**Figure 1:**
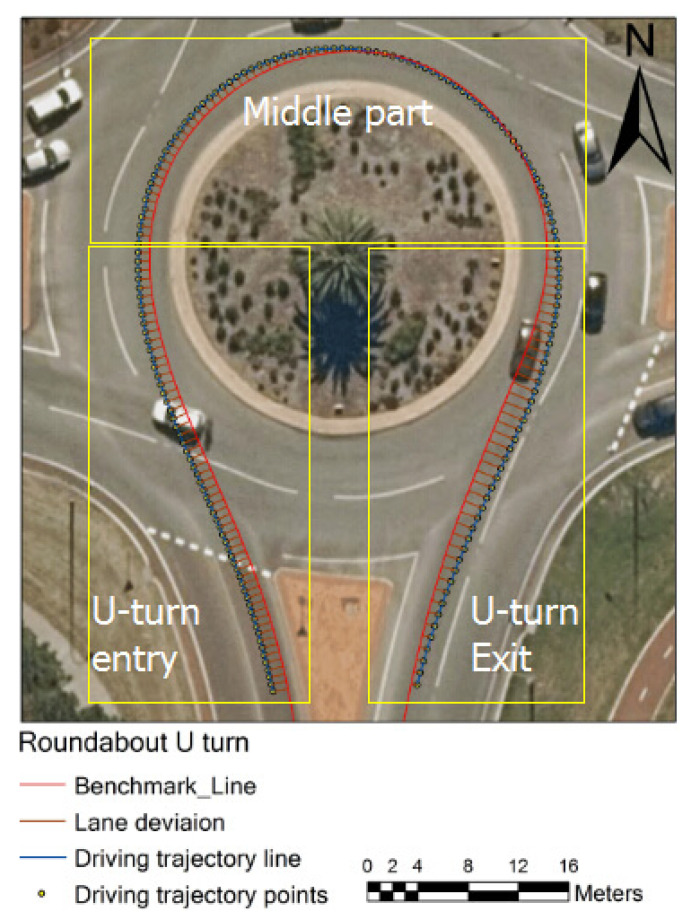
Driving trajectory and lane deviation from benchmark line in a U-turn and the three sections of the U-turn.

Lane keeping performance is a vital indicator of driving performance [17] and it is commonly measured by the standard deviation of lane deviation (SDLD), with lower standard deviation of lane deviation values indicating better lane keeping performance [17]. Lane deviation is defined as the perpendicular distance between the vehicle position and the lane centre line, whereas the smaller value of the standard deviation of lane deviation indicates better lane keeping performance (SDLD) [17,27]. Cao and Liu [17] suggested that lower driving speed can reduce the driver’s mean value of the standard deviation of lane deviation (SDLD). Their statistical analysis found that the mean value reduced from 0.36 to 0.30 m when the driving speed reduced from 72 to 36 km/h. In addition, a more difficult road geometry requires a more complex mental cognition workload and executive function, which may give rise to poor lane keeping performance. However, some studies also show that if the road geometry of a lane keeping task is quite simple, the required mental workload becomes very low, which could lead to a poor lane keeping performance due to a lack of excitement and motivation [28]. Therefore, this work focused on some of the more demanding parts of the on-road test’s driving route such as the roundabouts (see Figure 1) and left turns. Participants’ driving trajectories in simple driving tasks (straight driving) were also explored in this study.

#### 2.3.2. Speed Control

The capacity of speed control can also influence drivers’ driving safety. It is known that older and novice drivers are more likely to respond inappropriately to road geometries and traffic signs, which require them to control their driving speed [29]. Similarly, Weihong and Blythe [30] found that 80% of 60+ drivers did not control their speed appropriately in free-flow traffic conditions, and older drivers have been found to commit more driving errors when compared to younger drivers; however, their speed adjustment and lane position were improved in automatic transmission cars [31]. Furthermore, older drivers usually experience a high crash risk in urban environments, due to their inappropriate speed control performance [30]. Hence, the mean speed and standard deviation of speed can indicate the drivers’ speed control performance [32].

The foci of this study were lane maintenance, speed control and driving trajectory measured whilst undertaking a U-turn at a roundabout, a left turn and two straight line sections (as indicated in Appendix A). The driving trajectory of each participant consisted of a set of position points. The perpendicular distances between the points and the benchmark line were defined as the lane deviations. The perpendicular tool in ArcGIS software was applied to draw and calculate the distance of the perpendicular segments between the vehicle’s position points and the benchmark line [27].

**Figure 2:**
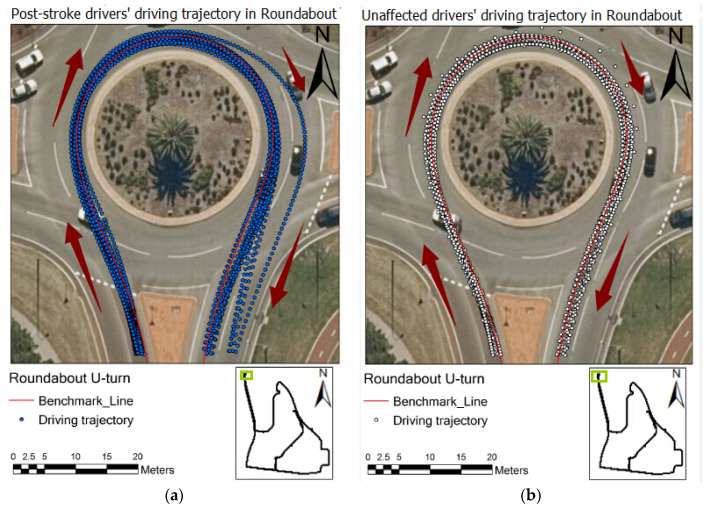
Driving trajectory of post-stroke (**a**) and normal (**b**) groups in a U-turn.

The SDLD and speed in each driving task (e.g., U-turn, left turn and straight line section) were also calculated. Figure 2 illustrates one example of a lane deviation (distance of the perpendicular segments) in a U-turn based on the perpendicular tool. Due to the geometry and complexity of the U-shaped roundabout, the roundabout was divided into three parts, entering U-turn/entry, middle part and exiting U-turn/exit (see Figure 2). The speed control performance for every participant was calculated by comparing the speed at position points with the known speed limits at each section. The speed limits at straight line one, U-turn and straight line two are 50, 40 and 70 km/h, respectively.

### 2.4. Statistical Analysis

Table 1 reports the statistical tests and the software used for analysing the data in this study. As Montella et al. [33] indicated, the lane keeping performance and speed data across post-stroke and normal older driver groups were tested for normality and homoscedasticity. The Shapiro–Wilk test was used to verify the normality assumption, whereby Levene’s test was applied to verify the homoscedasticity assumption. Python language [34] was used to carry out the verification tests. The testing results (reported in Appendix B) showed that the data across the two groups were normally distributed, and the within-group variances of the groups were equal in most cases. However, there were still some cases where the data did not pass the normality and homoscedasticity verification test, which hence required a non-parametric test. Therefore, this study not only applied parametric tests, but also the non-parametric test to more reliably examine the potential differences in lane keeping and speed control performance between the two groups.

Specifically, a two-tailed T-test using Python language and a one-way ANOVA test in SPSS version 21.0 [35] software were both applied to examine the differences in SDLD between the post-stroke group and normal comparison group in both complex (driving through a U-turn and left turn) and simple driving tasks (straight line driving). In addition to the parametric tests, the Wilcoxon rank-sum test (also named the Mann–Whitney U test) was also applied to investigate the significance of the potential differences. The critical α-value was set to 0.05 for all tests.

## 3. Results

The results were generated based on spatial and statistical analyses of the differences in lane keeping performance between the post-stroke and neurotypical older drivers.

### 3.1. Spatial Analyses of Driving Trajectories across the Groups

Figure 2 shows the post-stroke and normal drivers’ driving trajectories in the exit part of a U-turn. The post-stroke older drivers’ deviation from the road centre line in the U-turn exit was larger than that of normal older drivers. The recorded driving trajectory of the post-stroke older drivers did not cluster around the centre line of the road in the U-turn exit; furthermore, some participants merged to another lane during driving through the exit part of the U-turn. Comparatively, all the normal older drivers’ driving trajectories distributed more closely towards the benchmark line, as demonstrated in Figure 2, suggesting that the post-stroke drivers’ lane keeping performance in a relatively complex driving task was deemed poorer than that of normal drivers.

**Figure 3:**
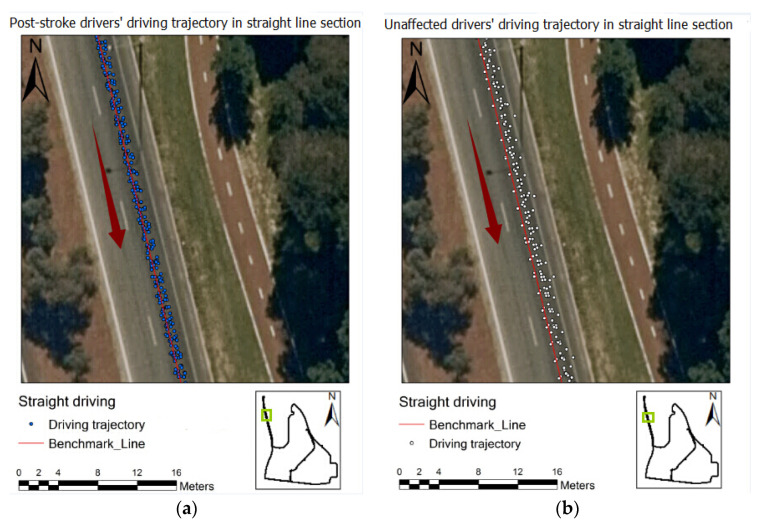
Driving trajectory of post-stroke (**a**) and normal (**b**) groups in a straight line.

**Figure 4:**
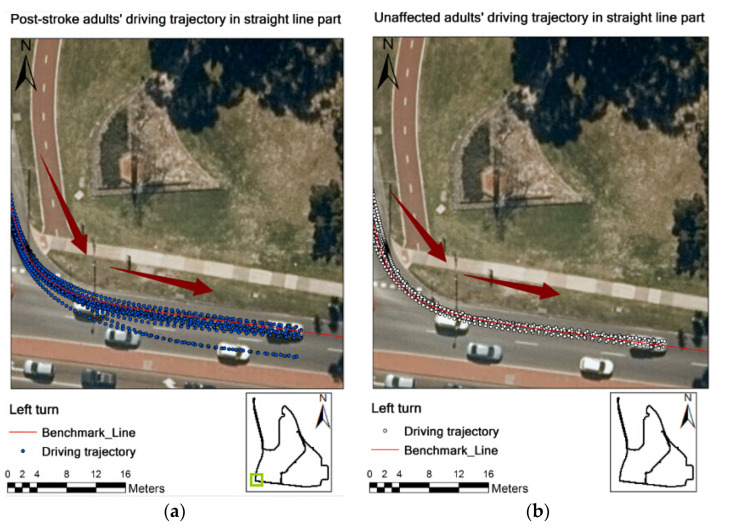
Driving trajectory of post-stroke (**a**) and normal (**b**) groups in left turn.

Figure 3 exhibits the post-stroke drivers’ and normal drivers’ driving trajectories in the straight line section of the on-road test. The blue points in Figure 4 represent the post-stroke older drivers’ driving trajectories, which were gathered on the centre line of the lane. Surprisingly, the distances (lane deviation) between the normal drivers’ driving trajectory and the benchmark line in Figure 4 were relatively larger than those of post-stroke drivers, indicating that the normal older drivers’ lane keeping performance in simple driving tasks may be actually poorer than that of the post-stoke drivers.

Figure 4 shows the post-stroke drivers’ and normal drivers’ driving trajectories in the left turn of the on-road driving test. The driving trajectories exhibit that the post-stroke drivers’ deviation from road centre line in the left turn was larger than that of normal drivers. In comparison with post-stroke older drivers’ driving trajectories, the normal older drivers’ driving trajectories distributed more closely towards the road centre line. Thus, Figure 4 indicates that the post-stroke drivers’ lane maintenance in left turns was deemed poorer than that of normal drivers.

### 3.2. Statistical Analyses of Driving Trajectories

#### 3.2.1. Roundabout (U-Turn)

As shown in Table 2, the mean SDLD values of the post-stroke group were generally higher than the values of the normal group. As outlined in Table 2, in the entire U-turn, the mean SDLD of the post-stroke group was 0.60 m, which was almost twice the value of the normal group; however, the *p*-values from the one-way ANOVA test, the *T*-test and the Wilcoxon rank-sum test were not significant. Similarly, the entry and middle part of the U-turn mean SDLD scores were generally higher; however, they were also not significant.

In the exit section of the U-turn, the value of the lane deviation in the post-stroke drivers was significantly larger than the control group’s lane deviation (1.38 m), where the *p*-values from the three tests are all less than 0.05. Furthermore, in the exit of the U-turn, the mean SDLD value of post-stroke participants was 0.48 m, which was twice that of the normal group of 0.19 m, with all *p*-values around 0.030, which suggests that the post-stroke participants’ lane keeping performance was statistically significantly poorer than that of normal drivers.

Table 2 also shows that in the U-turn exit, the post-stroke older drivers’ mean driving speed and standard deviation of speed (32.51, 5.17 km/h) are both higher than those (31.17, 4.79 km/h) of normal older drivers; however, differences were not statistically significant.

#### 3.2.2. Left Turn

As shown in Table 3, the mean lane deviation value of the post-stroke group was 0.52 m, which was higher than that of the normal group (0.37 m). This suggests that the normal older drivers had a smaller lane deviation from the benchmark line than the post-stroke older drivers; however, both parametric and non-parametric tests suggested that the differences in lane deviation were not statistically significant. The mean SDLD of the post-stroke group was 0.38 m, which is less than that of the normal group (0.20 m), suggesting that the post-stroke older drivers may perform more poorly, but again the *p*-value (0.106) was not significant.

#### 3.2.3. Straight Line One (Speed Limit of 50 km/h)

As Table 4 shows, in straight line section one (speed limit of 50 km/h), the post-stroke group’s mean lane deviation value was 0.65 m, which was smaller than that of the normal group (0.83 m), and the mean SDLD value of the post-stroke group was 0.20 m, which was also less than that of the normal group (0.28 m). Although these lower values show that the post-stroke older drivers maintained a more consistent line, the differences were again not significant.

The post-stroke group’s mean speed was faster than that of the normal group (48.16 and 45.00 km/h, respectively) and, similarly, the speed control performance measured by the standard deviation of speed was greater in the post-stroke group compared to the normal group (2.58 and 1.77 km/h, respectively); however, the differences were not significant.

#### 3.2.4. Straight Line Two (Speed Limit of 70 km/h)

The lane deviation and SDLD of the two groups in straight line section two (speed limit of 70 km/h) are summarised in Table 5. The mean lane deviation value of the post-stroke group was 0.30 m, which was half that of the normal group of 0.60 m, where the *p*-values from the ANOVA test, T-test and Wilcoxon rank-sum test are 0.015, 0.019 and 0.024 < 0.050. Furthermore, the post-stroke group’s mean SDLD value was 0.14 m, which was equal to that of the normal group.

The average speed and the standard deviation of speed in the post-stroke group (64.24, 1.68 km/h) were higher than those of the normal group (62.38, 1.34 km/h), but the *p*-values suggested that both differences were insignificant.

Generally, for the relatively complex driving tasks—U-turn and left turn—from the perspective of the size of the means, Table 2 and Table 3 show that post-stoke drivers underperformed the normal drivers, due to their relatively larger means of lane deviation and SDLD, with an exception at the entry of the roundabout where the normal drivers’ lane deviation means were slightly larger than those of post-stroke drivers. Additionally, in terms of the standard deviations (in parentheses after the mean), the post-stroke drivers normally had a relatively larger value in most cases (e.g., entire U-turn, middle part of U-turn and the exit), which suggests that the post-stroke drivers’ lane keeping performance is more likely to vary greatly from individual to individual.

On the contrary, for the relatively simple driving task of straight line driving, the comparison of the means and standard deviations between the two groups is inclusive, whereby the post-stroke drivers even have smaller lane deviation means and standard deviations. This interesting result suggests that post-stroke subjects could easily deal with the simple driving tasks, as these tasks may only demand a low mental workload. One explanation for the larger mean and standard deviation values of the normal drivers is that they were too negligent while driving through the straight line road sections.

## 4. Discussion

The underlying motivation of this pilot study was to assess the feasibility, appropriateness and effectiveness of using advanced GPS modelling as a tool to differentiate the driving performance of post-stroke drivers from that of normal older drivers. A subtle difference in driving competency is hard to detect using observation, but GNSS and RTK technologies enable accuracy of vehicle movement tracking and therefore provide detailed information on driving performance. The GNSS receiver can obtain the signal from multiple satellite systems. Additionally, RTK technology was applied after loading the trajectory data from GNSS receiver, which uses a known position of a base station to correct the recorded trajectory data and improves the data accuracy and precision to a high level of data accuracy (sub-decimetre level). Thus, the subtle difference in the participants’ driving trajectory and behaviour was explored, which indicated that the GNSS modelling was an appropriate and effective tool to explore on-road driving performance

Although multi-GNSS RTK for tracking driving behaviour has previously been utilised [22], as far as the authors are aware, this is the first study to investigate post-stroke driver performance on-road. The present study found that post-stroke adults performed more consistently in the straight line section and less consistently than the normal drivers in the exit to the roundabout. However, with the exception of the exit on the roundabout, there were no statistically significant differences in the speed maintenance or lane deviation performance between the post-stroke drivers and the normal adults. This would suggest that despite the differences in lane and speed deviation between groups, the post-stroke drivers were just as safe as the control participants. This aligns well with the fact that all the post-stroke adults had been cleared to drive by a medical professional.

A small sample size is one important limitation of this study, which increases the risk of a type II error; however, as the α value was set to 0.05, only statistical differences at the exit on the roundabout between two groups were found in the study. Further research with a greater sample size is required to address this.

The gender imbalance (3 females, 11 males) in the post-stroke group is another limitation that may affect the comparison of the post-stroke and normal groups, particularly as male post-stroke subjects are more likely to return to driving than females [36]. However, as volunteer sampling was employed, it was difficult to recruit enough participants to gender match. Further research with a greater sample and with gender matching would address this problem.

For the on-road driving test, each participant performed the driving test using his/her own vehicle. It is possible that this may have influenced their lane keeping and driving trajectory due to the tracking quality and vehicle transmission; however, it can be argued that participants’ performance was likely to be most valid in a vehicle with which they were familiar. Further, as some participants required vehicle alterations (e.g., a spinner knob for hemiplegia or hemiparesis) for safety and insurance reasons, it was decided that all participants were to use to their own vehicle.

Although the same fixed route was applied for each participant in order to minimise the effects of road changes, one of the major difficulties in on-road testing is that the road conditions are likely to change between assessments, e.g., traffic or weather. In order to control for this as much as possible, participants were assessed between the hours of 10.00 a.m. and 4.00 p.m. to avoid peak hour congestion and assessors noted whether there was any significant change in the driving route, e.g., due to bad weather, road works or traffic accidents.

## 5. Conclusions

It can be concluded that lane keeping might be an indicator of driving performance when driving at a U-turn, particularly exiting the roundabout (complex driving task), while speed control might be superior in revealing driving performance in a straight line. Cognitively demanding driving situations, such as U-turns and particularly exiting the roundabout, create a challenge for post-stroke older drivers. This may be ascribed to the higher levels of cognitive abilities required to maintain the lane along the changing geometry of the road [17]. For understanding the association between lane keeping, speed maintenance and cognitive abilities, especially divided and selective attention requires further research that might explain why some post-stroke drivers’ mean SDLD stays relatively high.

The findings of this study demonstrate the appropriateness, feasibility and effectiveness of assessing driving behaviours of post-stroke older drivers. It is strongly recommended that SDLD calculated from an accurate vehicle movement trajectory is a sensitive and effective measure for driving assessment in this cohort population. Future work will need to examine and model older drivers’ lane keeping and speed regulation in the face of hazardous driving situations. Further educational and training programs based on the findings of this study could be developed to enhance post-stroke older drivers’ behaviour behind the wheel; for example, neuropsychological training to improve post-stroke older drivers’ executive function [37]; and driving intervention training to improve lane keeping performance [38] at challenging driving sections.

## Figures and Tables

**Table 1:** Tests and software.

Statistical Test		Software	Data	Sample Size	Purpose
Normality test	Kolmogorov–Smirnov test	SPSS 21.0	Post-stroke and normal older driver groups	14 participants in each group	To check the normality of the sample data
Parametric test	Two-tailed *T*-test	Python statistical analysis package			To exam the potential differences in lane keeping and speed control performance between the groups
	One-way ANOVA test	SPSS 21.0			
Non-Parametric test	Wilcoxon rank-sum test	Python statistical analysis package			

**Table 2:** Statistical tests of lane keeping performance and speed control in U-turn.

Variable ^1^	Group	Mean (std)	Median	IQR ^2^	One-Way ANOVA Test	Two-Tailed *T*-Test	Wilcoxon Rank-Sum Test
Lane deviation (Entire U-turn)	Post-stroke	0.78 (0.48)	0.67	0.42	0.144	0.148	0.129
	Normal	0.55 (0.26)	0.49	0.14			
SDLD (Entire U-turn)	Post-stroke	0.60 (0.48)	0.45	0.42	0.055	0.064	0.081
	Normal	0.32 (0.11)	0.36	0.09			
Lane deviation (Entry of U-turn)	Post-stroke	0.57 (0.27)	0.49	0.47	0.766	0.766	0.783
	Normal	0.61 (0.35)	0.76	0.67			
SDLD (Entry of U-turn)	Post-stroke	0.23 (0.12)	0.21	0.14	0.872	0.872	0.927
	Normal	0.22 (0.10)	0.23	0.19			
Lane deviation (Middle part of U-turn)	Post-stroke	0.55 (0.36)	0.45	0.28	0.544	0.544	0.183
	Normal	0.46 (0.40)	0.30	0.50			
SDLD (Middle part of U-turn)	Post-stroke	0.29 (0.29)	0.19	0.14	0.194	0.201	0.168
	Normal	0.18 (0.10)	0.16	0.11			
Lane deviation (Exit of U-turn)	Post-stroke	1.38 (1.24)	1.05	0.75	0.043 *	0.049 *	0.035 *
	Normal	0.62 (0.37)	0.63	0.68			
SDLD (Exit of U-turn)	Post-stroke	0.48 (0.43)	0.31	0.35	0.026 *	0.032 *	0.027 *
	Normal	0.19 (0.11)	0.19	0.14			
Driving speed (km/h) (Exit of U-turn)	Post-stroke	32.51 (3.06)	31.27	5.35	0.634	0.635	0.783
	Normal	31.71 (5.17)	32.53	3.50			
Std. deviation of speed (km/h) (Exit of U-turn)	Post-stroke	5.17 (1.20)	5.06	1.80	0.348	0.350	0.370
	Normal	4.79 (0.81)	4.60	1.08			

^1^ mean in metres; ^2^ interquartile range (IQR) = Q3 − Q1. *: *p*-value < 0.05.

**Table 3:** Statistical tests of lane keeping performance in left turn.

Variable	Group	Mean (std)	Median	IQR	One-Way ANOVA Test	Two-Tailed *T*-Test	Wilcoxon Rank-Sum Test
Lane deviation (Left turn)	Post-stroke	0.52 (0.31)	0.46	0.24	0.154	0.158	0.198
	Normal	0.37 (0.16)	0.37	0.17			
SDLD (Left turn)	Post-stroke	0.38 (0.39)	0.23	0.16	0.106	0.117	0.291
	Normal	0.20 (0.06)	0.22	0.08			

**Table 4:** Statistical tests of lane keeping performance and speed control in straight line section one.

Variable	Group	Mean (std)	Median	IQR	One-Way ANOVA Test	Two-Tailed *T*-Test	Wilcoxon Rank-Sum Test
Lane deviation (Straight line)	Post-stroke	0.65 (0.26)	0.68	0.34	0.104	0.104	0.118
	Normal	0.83 (0.29)	0.81	0.54			
SDLD (Straight line)	Post-stroke	0.24 (0.08)	0.24	0.07	0.330	0.330	0.408
	Normal	0.28 (0.11)	0.26	0.13			
Driving speed (km/h)	Post-stroke	48.16 (6.72)	49.33	6.85	0.285	0.286	0.198
	Normal	45.00 (7.97)	45.79	5.91			
Std. deviation of speed (km/h)	Post-stroke	2.58 (1.78)	1.83	1.55	0.163	0.166	0.270
	Normal	1.77 (0.96)	1.75	1.20			

**Table 5:** Statistical tests of lane keeping and speed control in straight line section two.

Variable	Group	Mean (std)	Median	IQR	One-Way ANOVA Test	Two-Tailed *T*-Test	Wilcoxon Rank-Sum Test
Lane deviation (Straight line)	Post-stroke	0.30 (0.15)	0.26	0.25	0.015 *	0.019 *	0.024 *
	Normal	0.60 (0.39)	0.56	0.41			
SDLD (Straight line)	Post-stroke	0.14 (0.09)	0.12	0.06	0.826	0.826	0.646
	Normal	0.14 (0.08)	0.12	0.07			
Driving speed (km/h)	Post-stroke	64.24 (6.04)	63.66	8.98	0.521	0.521	0.963
	Normal	62.38 (8.49)	63.34	7.78			
Std. deviation of speed (km/h)	Post-stroke	1.68 (0.74)	1.41	1.34	0.307	0.307	0.141
	Normal	1.34 (0.91)	1.07	1.67			

*: *p*-value < 0.05.

**Figure A1:**
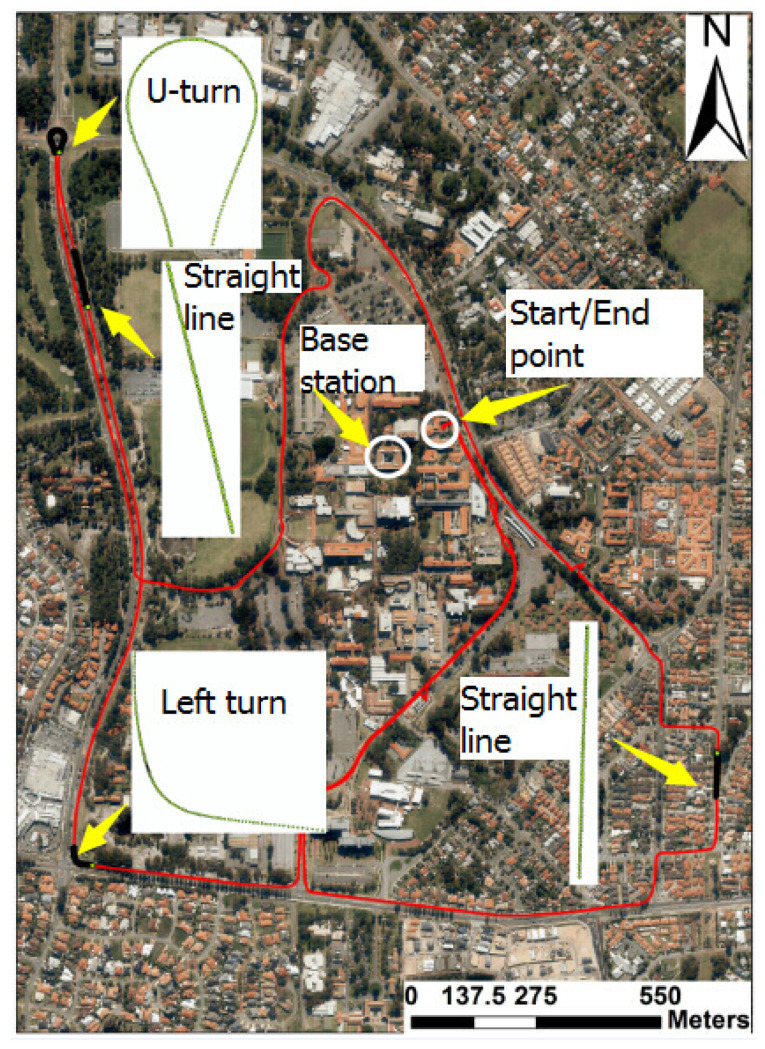
The on-road driving assessment route with focus junctions and base stations indicated.

## Data Availability

The data presented in this study are available on request from the corresponding author. The data are not publicly available due to ethical and privacy restrictions.

## Appendix B

**Table A1:** Normality and homoscedasticity tests.

Variable	Group		Shapiro–Wilk Test		Levene’s Test
Lane deviation (Entire U-turn)	Post-stroke	Normal	0.001	0.002	0.309
SDLD (Entire U-turn)	Post-stroke	Normal	0.001	0.015	0.058
Lane deviation (Entry of U-turn)	Post-stroke	Normal	0.405	0.065	0.365
SDLD (Entry of U-turn)	Post-stroke	Normal	0.041	0.198	0.896
Lane deviation (Middle part of U-turn)	Post-stroke	Normal	0.002	0.005	0.664
SDLD (Middle part of U-turn)	Post-stroke	Normal	0.000	0.141	0.396
Lane deviation (Exit of U-turn)	Post-stroke	Normal	0.001	0.209	0.130
SDLD (Exit of U-turn)	Post-stroke	Normal	0.002	0.266	0.036
Driving speed (Exit of U-turn)	Post-stroke	Normal	0.099	0.000	0.766
Std. deviation of speed (Exit of U-turn)	Post-stroke	Normal	0.700	0.495	0.214
Lane deviation (Left turn)	Post-stroke	Normal	0.004	0.971	0.329
SDLD (Left turn)	Post-stroke	Normal	0.000	0.542	0.077
Lane deviation (Straight line)	Post-stroke	Normal	0.827	0.154	0.639
SDLD (Straight line)	Post-stroke	Normal	0.676	0.140	0.522
Driving speed	Post-stroke	Normal	0.327	0.039	0.854
Std. deviation of speed	Post-stroke	Normal	0.002	0.154	0.411
Lane deviation (Straight line)	Post-stroke	Normal	0.412	0.222	0.022
SDLD (Straight line)	Post-stroke	Normal	0.004	0.044	0.879
Driving speed	Post-stroke	Normal	0.653	0.000	0.881
Std. deviation of speed	Post-stroke	Normal	0.032	0.002	0.977
